# Characterisation of Fasting and Postprandial NMR Metabolites: Insights from the ZOE PREDICT 1 Study

**DOI:** 10.3390/nu15112638

**Published:** 2023-06-05

**Authors:** Kate M. Bermingham, Mohsen Mazidi, Paul W. Franks, Tyler Maher, Ana M. Valdes, Inbar Linenberg, Jonathan Wolf, George Hadjigeorgiou, Tim D. Spector, Cristina Menni, Jose M. Ordovas, Sarah E. Berry, Wendy L. Hall

**Affiliations:** 1 Department of Nutritional Sciences, King’s College London, London WC2R 2LS, UKwendy.hall@kcl.ac.uk (W.L.H.); 2 Department of Twins Research and Genetic Epidemiology, King’s College London, London WC2R 2LS, UK; 3 Medical Research Council Population Health Research Unit, University of Oxford, Oxford OX1 3QR, UK; 4 Clinical Trial Service Unit and Epidemiological Studies Unit (CTSU), Nuffield Department of Population Health, University of Oxford, Oxford OX3 7LF, UK; 5 Department of Clinical Sciences, Lund University, 21428 Malmö, Sweden; 6 Department of Nutrition, Harvard T.H. Chan School of Public Health, Boston, MA 02215, USA; 7 School of Medicine, University of Nottingham, Nottingham NG5 1PB, UK; ana.valdes@nottingham.ac.uk; 8 Nottingham NIHR Biomedical Research Centre, Nottingham NG7 2UH, UK; 9 ZOE Ltd., London SE1 7RW, UKgeorge@joinzoe.com (G.H.); 10 Jean Mayer USDA Human Nutrition Research Centre on Aging (JM-USDA-HNRCA), Tufts University, Boston, MA 02111, USA; jose.ordovas@tufts.edu; 11 IMDEA Food Institute, CEI UAM + CSIC, 28049 Madrid, Spain; 12 Centro de Investigación Biomédica en Red-Fisiopatología de la Obesidad y Nutrición (CIBEROBN), Instituto de Salud Carlos III, 28029 Madrid, Spain

**Keywords:** lipids, lipoproteins, nuclear magnetic resonance (NMR)

## Abstract

Background: Postprandial metabolomic profiles and their inter-individual variability are not well characterised. Here, we describe postprandial metabolite changes, their correlations with fasting values and their inter- and intra-individual variability, following a standardised meal in the ZOE PREDICT 1 cohort. Methods: In the ZOE PREDICT 1 study (*n* = 1002 (NCT03479866)), 250 metabolites, mainly lipids, were measured by a Nightingale NMR panel in fasting and postprandial (4 and 6 h after a 3.7 MJ mixed nutrient meal, with a second 2.2 MJ mixed nutrient meal at 4 h) serum samples. For each metabolite, inter- and intra-individual variability over time was evaluated using linear mixed modelling and intraclass correlation coefficients (ICC) were calculated. Results: Postprandially, 85% (of 250 metabolites) significantly changed from fasting at 6 h (47% increased, 53% decreased; Kruskal–Wallis), with 37 measures increasing by >25% and 14 increasing by >50%. The largest changes were observed in very large lipoprotein particles and ketone bodies. Seventy-one percent of circulating metabolites were strongly correlated (Spearman’s rho >0.80) between fasting and postprandial timepoints, and 5% were weakly correlated (rho <0.50). The median ICC of the 250 metabolites was 0.91 (range 0.08–0.99). The lowest ICCs (ICC <0.40, 4% of measures) were found for glucose, pyruvate, ketone bodies (β-hydroxybutyrate, acetoacetate, acetate) and lactate. Conclusions: In this large-scale postprandial metabolomic study, circulating metabolites were highly variable between individuals following sequential mixed meals. Findings suggest that a meal challenge may yield postprandial responses divergent from fasting measures, specifically for glycolysis, essential amino acid, ketone body and lipoprotein size metabolites.

## 1. Introduction

Advancements in metabolomics and the development of comprehensive high-throughput profiling have enabled the simultaneous quantification of multiple biomarkers in large cohorts [1,2,3,4]. This has progressed our understanding of the mechanistic pathways linking metabolites to disease risk and enabled early identification of elevated risk for early atherosclerosis, type 2 diabetes, diabetic nephropathy, cardiovascular diseases and all-cause mortality [5]. To date, metabolomic profiles have been reported mainly in the fasting state [1]. However, the physiological relevance of fasting analyses is a point of debate [6,7], since we consume multiple mixed-nutrient meals throughout the day, and therefore spend most of our time in the highly dynamic postprandial state.

Moreover, postprandial metabolic dysregulation is an independent risk factor for non-communicable diseases [8,9,10], but the relevance for health of non-standard meal-induced postprandial metabolomic markers is less clear. Standard clinical biochemistry analysis of blood glucose, triglycerides (TG) and insulin alone does not fully represent the multiple downstream postprandial metabolic changes that can be captured from metabolomic analysis and potentially harnessed for improved sensitivity in the prediction of pre-clinical risk of cardiometabolic diseases. To date, studies examining postprandial metabolomics have been conducted in small cohorts [11] or have focused on specific metabolites, instead of quantifying a broad range of metabolomic responses [12,13]. Furthermore, despite growing awareness of the large inter-individual variability in metabolic responses to food [14], this has rarely been explored beyond simple clinical measures.

Given that metabolomic profiles and postprandial metabolic dysregulation are established independent risk factors for disease risk [5], improved understanding of postprandial metabolomic responses to food is necessary to inform understanding of the relationship between diet and health. The ZOE PREDICT 1 study was designed to quantify and predict individual variations in fasting and postprandial TG, glucose and insulin responses to sequential standardised meals in a tightly controlled setting [14]. The aim of this study was to explore and compare inter-individual fasting and postprandial variabilities in metabolomic profiles.

## 2. Materials and Methods

The ZOE PREDICT 1 study (NCT03479866) was a single-arm, single-blinded study (June 2018 to May 2019) in 1102 healthy adults, aged 18–65 y (*n* = 1002 from the United Kingdom (UK); for the full protocol, see Berry et al. [15]. The study was conducted between 5 June 2018 and 8 May 2019, with participants recruited from the TwinsUK cohort, an ongoing research cohort described elsewhere [16] and through online advertising. The study consisted of a 1-day clinical visit at baseline followed by a 13-day at-home period, although this paper only focuses on the 1-day clinical visit. Primary outcomes are reported elsewhere [13,14]. Secondary outcome metabolomic data measured by NMR (at the baseline visit only) was reported previously [17] and in this paper. At baseline (day 0), participants arrived fasted and were given a standardised metabolic challenge meal for breakfast (0 h; 86 g carbohydrate, 53 g fat, 16 g protein; 3.7 MJ) and a test lunch (4 h; 71 g carbohydrate, 22 g fat, 10 g protein; 2.2 MJ). The fat was high oleic sunflower oil; 85% oleic acid (18:1n − 9) and 8% linoleic acid (18:2n − 6). Fasting and postprandial (0–6 h) venous blood was collected to determine concentrations of serum glucose, insulin, TG and metabolomics (using NMR described below). The trial was approved in the UK by the Research Ethics Committee and Integrated Research Application System (IRAS 236407), registered on ClinicalTrials.gov (NCT03479866) and was run in accordance with the Declaration of Helsinki and Good Clinical Practice. Participants provided informed written consent before taking part in the study and individual participants could not be identified following data collection.

Metabolite Measurements: Metabolite concentrations were quantified at three time points from serum at fasting, 4 h and 6 h postprandially using high-throughput NMR metabolomics (2020 Platform; Nightingale Health, Helsinki, Finland). The metabolomics platform provides 250 parameters (concentrations, ratios, size, percentages) derived from 163 raw metabolite measures (concentrations and size). Details of the experimentation and epidemiological applications of the NMR metabolomics platform have been reviewed previously [18].

Diet Assessment: Participants completed the validated European Prospective Investigation into Cancer and Nutrition (EPIC) Food-Frequency Questionnaire (FFQ), which is used to measure habitual food and nutrient intakes over the past year. FETA software was used to calculate nutrient data [19] and the Healthy Eating Index (HEI) score was calculated as a measure of diet quality [20]. Data were excluded if the total energy intake estimate, calculated from the FFQ as a ratio of the subject’s estimated basal metabolic rate (determined by the Harris–Benedict equation), was more than 2 SD outside the population mean for this ratio (<0.52 or >2.58), or if more than ten items of the FFQ were left unanswered, as previously described [14].

Statistical Analysis: Statistical analysis was performed in the R environment for statistical computing version 3.5.1 (R Foundation for Statistical Computing, Vienna, Austria. https://www.R-project.org/, accessed on 11 April 2023). Metabolites were characterised by mean, median, 25th and 75th percentiles at fasting and 4 h and 6 h postprandially. Absolute change and percentage change were calculated. Kruskal–Wallis tests were performed to evaluate differences in the median concentrations at fasting and 4 h and fasting and 6 h. Spearman’s correlation assessed the relationship between measures at fasting and 4 h/6 h and the Fligner–Killeen test compared variances at fasting and 4 h/6 h. Spearman’s correlations also assessed associations between 6 h absolute change and diet quality (HEI), BMI, fasting glucose and age. Time-dependent changes in metabolite concentrations within individuals were evaluated using mixed models. Total variance in plasma metabolites was decomposed into inter-individual variance, which can also be considered the variance of the usual level in a population, and intra-individual variance, which reflects variability around the usual level within an individual. Fasting and postprandial metabolite levels were included as the outcome variables, with time as a fixed effect and participant ID as a random effect. Intraclass correlations (ICC) were calculated, denoting the proportion of the population’s biologic variability that is due to the inter-individual variation [21,22]. A high ICC can be obtained by low intra- and/or high inter-individual variance. A low ICC is attributable to high intra- and/or low inter-individual variance. The Benjamini–Hochberg correction for multiple comparisons was applied [23]. Changes in lipoprotein subclass particle concentrations that occurred during the mixed meal challenge were assessed in males and females separately, using repeated-measures ANOVA. Statistically significant thresholds were based on FDR cut-offs (*q* < 0.05). Figures were made using Prism, Version 9.2.0.

## 3. Results

**Figure 1:**
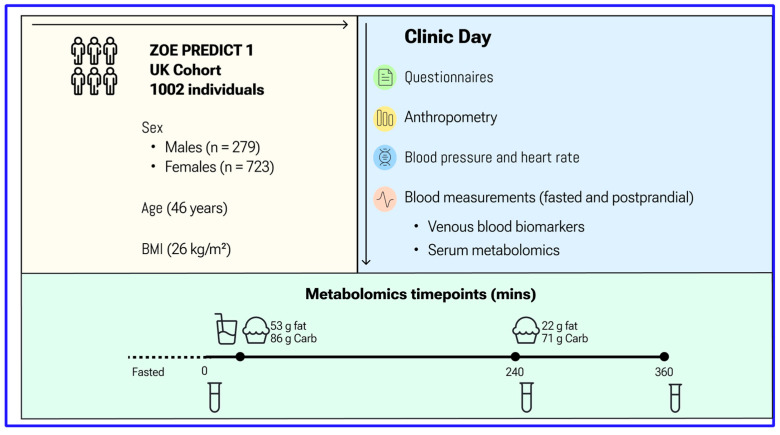
ZOE PREDICT 1 Study Design. Participants arrived fasted for their baseline visit and were given a standardised breakfast (0 h, metabolic challenge meal, 86 g carbohydrate, 53 g fat) and lunch (4 h, 71 g carbohydrate, 22 g fat). Concentrations of glucose, TG and NMR metabolites were determined from venous blood collected at multiple timepoints postprandially. Anthropometric and fasting biochemistry measurements were also measured.

A total of 1002 generally healthy adults completed baseline (day 0) measurements and the sequential test meal challenge. Descriptive characteristics of study participants are summarised in Supplementary Table S1 and the study design is shown in Figure 1. Participants were aged between 18.5 and 65.9 (mean 45.6 ± 11.9) years, with a mean BMI of 25.6 (±5.0) kg/m^2^.

Characterization of Metabolite Biomarkers: Metabolite concentrations measured as mean, median and IQR are reported in Supplementary Table S2 for fasting, 4 and 6 h values for 250 metabolites. A selection of metabolites (*n* = 75) is also presented in Table 1.

**Figure 2:**
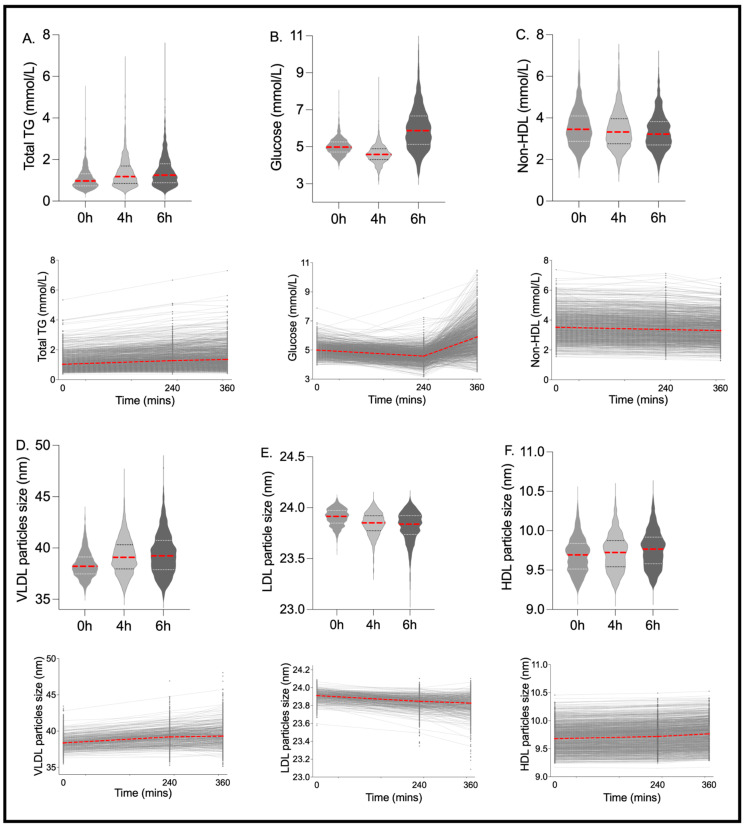
Inter-individual variation and distribution for traditional clinical metabolites and lipoprotein particle size. Fasting and postprandial concentrations of (**A**) triglycerides (TG) (mmol/L), (**B**) glucose (mmol/L), (**C**) non-high-density lipoprotein (HDL) (mmol/L) and particle sizes of: (**D**) very low-density lipoprotein (VLDL) (nm), (**E**) low-density lipoprotein (LDL) (nm), (**F**) high-density lipoprotein (HDL) (nm). *n* = 1002. Red lines show the median value.

**Figure 3:**
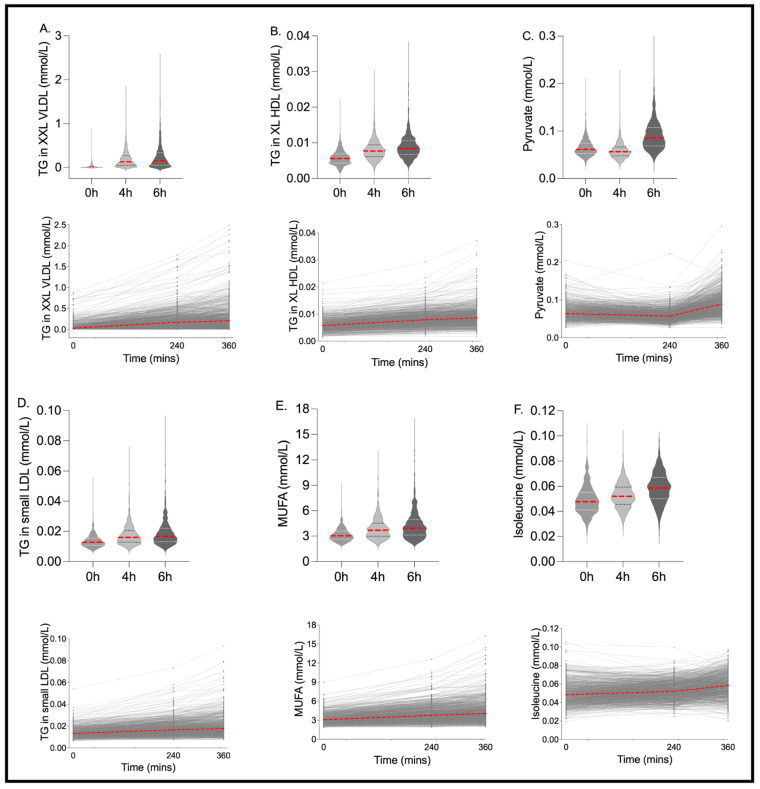
Metabolites with the greatest postprandial change and/or postprandial inter-individual variability. Fasting and postprandial concentrations of (**A**) triglycerides in extremely large VLDL particles and chylomicrons (TG in XXL VLDL), (**B**) triglycerides in large HDL particles and chylomicrons (TG in XL HDL), (**C**) triglycerides in LDL particles and chylomicrons (TG in LDL), (**D**) pyruvate, (**E**) mono-unsaturated fatty acids (MUFA), (**F**) isoleucine. *n* = 1002. Red lines show the median value.

Postprandial Change: Postprandially, 83% of the 250 metabolite outcomes measured had a significant absolute change at 4 h from fasting (43% with a significant increase and 57% with a significant decrease; Kruskal–Wallis FDR < 0.05), and 85% had a significant absolute change at 6 h from fasting (47% with a significant increase and 53% with a significant decrease; Kruskal–Wallis FDR < 0.05). The majority (95%) of those with a significant change at 4 h were also significantly changed at 6 h, while 8% of those that changed at 6 h were not different between fasting and the 4 h postprandial timepoints. At 6 h, 37 of the 250 metabolites changed by >25% from fasting values (30 increased and seven decreased by >25%), of which 14 changed by >50% from fasting values (12 increased and two decreased by >50%) (Supplementary Table S2; median % change). The largest postprandial increases (median % change; 0–6 h) were elicited in the XXL-VLDL particles, specifically particle number (XXL-VLDL-P; 440%), TG (XXL-VLDL-TG; 676%), phospholipid (XXL-VLDL-PL; 570%) and total lipid (XXL-VLDL-L; 379%) concentrations. The largest postprandial decreases (median % change; 0–6 h) were observed in ketone bodies (β-hydroxybutyrate: −85%, acetoacetate: −49%, acetate: −40%, acetone: −400%) and the percentage contribution of cholesterol (esters (CE) and total (C)) to XL-VLDL (XL-VLDL-CE: −57%, XL-VLDL-C: −46%). Traditional clinical measures (TG, glucose and non-HDL), lipoprotein particle sizes (due to their strong association with disease risk) and the variables with the largest postprandial change (>25%; 0–6 h), within each class of metabolite, are shown in Figure 2 and Figure 3.

Correlation between Fasting and Postprandial Metabolites: Postprandial concentrations of key food-induced metabolic markers, glucose and TG, are known to be more discriminatory of CVD risk than their fasting values [8,9,10]. However, if postprandial metabolites are closely correlated to their fasting values, there is minimal utility in conducting burdensome postprandial studies. Therefore, we assessed the correlation between fasting and postprandial measures to explore the value of measuring non-standard clinical measures postprandially. For most measures, the 4 h and 6 h values were strongly correlated with fasting values (Spearman’s rank correlation coefficient >0.80 in 80% (fasting 4 h) and 71% (fasting 6 h) of measures (Supplementary Table S2)). However, low correlations (rho < 0.50) were observed for ~5% of measures at both postprandial timepoints, including ketone bodies (β-hydroxybutyrate, acetate, acetoacetate), as well as glucose, pyruvate and lactate. LDL diameter, isoleucine and phenylalanine were also <0.50 between fasting and 6 h only. The lack of correlation for these measures may be due to significant variation within individuals (differences from one time point to another).

We also examined associations between postprandial metabolomic change (0–6 h) and factors including diet quality (HEI), BMI, fasting glucose and age (Supplementary Table S2). BMI had the strongest correlations (rho range; 0.00–0.36) and was correlated with the largest proportion of metabolites (82%) compared to glucose, age and diet quality. BMI was most strongly correlated with large VLDL metabolites and was not correlated with any large HDL or large LDL metabolites. Fasting glucose (rho range; 0.00–0.29) showed similar patterns of correlation to BMI. Age (rho range; 0.00–0.26) was most strongly correlated with TGs in the lipoprotein subclasses and, in addition, VLDL related metabolites. Correlations with an index of diet quality (HEI) were weaker (rho range; 0.00–0.14), but the top associations included cholesterol and cholesterol esters in medium and large VLDL metabolites, as well as some amino acids (phenylalanine and alanine).

Interindividual Variability in Metabolites Over Time: Given the highly variable postprandial responses observed in traditional clinical measures (TG, glucose and insulin) following a standardised meal in healthy individuals [15], we explored the variability in postprandial metabolomic responses. The proportion of total variability attributable to between-subject differences, as determined by the ICC (ratio of between-person variance and total variance (sum of intra- and inter-individual variances)), was high for most metabolites (ICC ≥ 0.75; 83%, 0.51–0.74; 12%, 0.40–0.50; 1%, <0.40; 4%). The median ICC of the 250 metabolites was 0.91 (range 0.08–0.99). The metabolites with the highest ranked ICCs included HDL measures (cholesterol, cholesterol esters, particle concentration, total lipids, phospholipids and free cholesterol in very large and large HDL), apolipoprotein B, the ratio of apolipoprotein B to apolipoprotein A and concentration of LDL particles. A selection of metabolites had lower ICCs (<0.40), meaning variation around an individual’s usual level was larger. These metabolites included glucose, pyruvate, ketone bodies (β-hydroxybutyrate, acetoacetate, acetate) and lactate.

The inter-individual pattern of response for fasting and 6 h time points was also assessed using the Fligner–Killeen test of variance. There were large differences in the variance of the data at 6 h versus fasting (Fligner–Killeen test of variance *p* < 0.001 for 39% of measures; *p* < 0.01 for 49% of measures; *p* < 0.05 for 58% of measures, Supplementary Table S2), illustrating the differential variability (spread) in the postprandial versus fasting state (Figure 2 and Figure 3).

Lipoprotein Subclass Concentrations Across Sexes: Male participants had higher particle concentrations of all VLDL particles, apart from very small VLDL particle concentrations, which were slightly higher in females postprandially (4 h; *p* = 0.048, 6 h *p* < 0.001) (Supplementary Table S3). Additionally, males and females displayed similar patterns of change in LDL particle concentrations, with decreases in large and medium particles. There were no differences in IDL particles across sexes. HDL particle concentrations were higher in females at all timepoints compared to males, except for small HDL particles, which were similar in the fasted state but postprandial concentrations became different (4 h; *p* < 0.001, 6 h; *p* < 0.001). Very large and large HDL particle concentrations increased over time while medium and small particles decreased. The magnitudes of these patterns (sex × time interaction) differed for all metabolites apart from medium HDL particle concentrations.

## 4. Discussion

This study set out to describe meal-induced changes in metabolomic markers, and to compare fasting and postprandial correlations and differences in inter-individual variability. Most non-traditional clinical metabolites from the Nightingale NMR panel showed large inter-individual variability following a mixed challenge meal. Greater inter-individual variability was observed in traditional clinical postprandial measures relative to equivalent fasting measures. A lack of correlation over time between fasting and postprandial metabolite concentrations was due to significant variation within individuals (differences from one time point to another). These findings suggest that postprandial responses for glycolysis, essential amino acid, ketone body and lipoprotein size metabolites may provide further insight into disease risk than fasting measures alone.

Research demonstrates that certain metabolites vary within an individual; for example, plasma 1H NMR metabolites vary during the menstrual cycle within pre-menopausal females [24]. Others are more stable over longer periods of time, reflecting the usual levels necessary for large-scale epidemiological research. The reliability over time of fasting blood metabolites has been investigated [19,20,25] and reliability over time has been shown to decrease in non-fasting samples [26]. Food intake influences the metabolomic profile, but short-term postprandial metabolomic responses, specifically in lipids and their subclasses, are less understood. This research shows that a meal challenge yielded lower ICCs in glycolysis, essential amino acid, ketone body and lipoprotein size metabolites. These measures have higher intra-individual variability and, thus, may provide more insight into divergent metabolic responses and associated disease risk. Most fasting and postprandial metabolites measured by the Nightingale NMR panel, mainly lipids and their subclasses, were shown to be stable in the postprandial response phase. Thus, analysis by more comprehensive metabolic panels may reveal postprandial perturbations not detected in this panel.

The postprandial changes in lipoprotein and lipid profiles, as well as significant inter-individual variability in postprandial responses, have been previously described [27,28,29,30,31,32]. Our findings showed postprandial percentage changes were greatest in the VLDL parameters, particularly in the concentration and lipids of the largest VLDL particles, in agreement with previous studies [29,32], and likely a marker of exogenous TG. The pattern of lipoprotein particle change observed for both men and women was also similar, although the magnitude of these changes appeared greater in men compared to women for VLDL response, similar to previous findings [29]. The metabolomic composition of plasma has also been shown to be affected by many factors [19,26,33,34] and this study demonstrates associations between postprandial metabolite change and age, sex, BMI and diet. Previous studies [27,28,29,30,31,32] have tested postprandial responses to a single meal. However, in real-life settings people generally consume multiple meals a day, with the carry-over effects of lipids and glucose metabolism evident at the sequential meal [35,36,37]. Therefore, based upon these findings, examining two consecutive mixed meals may better reflect the unfasted postprandial state.

TG-rich lipoproteins, chylomicrons, VLDL and their remnants (all captured in this platform under the VLDL particles), increase in the circulation following a fatty meal and are known to be atherogenic, with non-fasting TG concentrations being strongly associated with risk of CHD, stroke and mortality [37], and non-fasting small and large VLDL-C accounting for a 40% increased risk of myocardial infarction, associated with higher BMI [38]. Elevated postprandial TG concentrations that persist for up to 6 h and beyond are mainly attributable to greater increases in subclasses of large VLDL [39]. Our previous work has shown that using postprandial plasma TG concentrations as an indicator of the atherogenic potential of different meals may be misleading, since a saturated fat-rich meal induced lower postprandial TG concentrations but higher large VLDL concentrations at 6–8 h, compared with a monounsaturated-rich oil [40]. Therefore, quantifying postprandial large VLDL particles and particle composition may be more discriminatory than total TGs when assessing the atherogenic potential of a meal or food and their implications for CVD risk.

The strengths of this study are the large study population, repeated postprandial timepoints allowing analysis at peak lipemia and the later postprandial phase, and the design of the postprandial challenge, which adopted physiologically relevant macronutrient profiles and sequential meals. However, limitations of the study include a lack of longer postprandial follow-up (up to 8–10 h), which may have revealed NMR measures that were more discriminatory of metabolic status, and the limited panel of metabolites measured, which were predominantly lipids. Furthermore, we could not partition technical and intra-individual variability across timepoints. Nightingale NMR lipoprotein subclass profiling was recently challenged by Krauss et al. [41]; however, an updated biomarker quantification library was used for the current research, rendering the concerns regarding apolipoprotein B and particle numbers irrelevant. The consistency of the Nightingale biomarker measures in relation to other lab assays and disease endpoints have also been demonstrated in several publications [42,43,44].

In conclusion, this paper provides a large, comprehensive NMR spectroscopy metabolomics resource for lipid and postprandial metabolic research, and demonstrates that postprandial responses for glycolysis, essential amino acid, ketone body and lipoprotein size plasma metabolites may provide more insight into favourable metabolic responses and associated disease risk than fasting measures alone.

## Figures and Tables

**Table 1:** Characterization of concentrations of the metabolomic markers.

	Fasting			4 h			6 h			Fasting-6 h	
	Median	25th	75th	Median ^1^	25th	75th	Median ^1^	25th	75th	*p*-Value ^2^	ICC (95% CI)
Cholesterol											
Clinical LDL Cholesterol (mmol/L)	2.842	2.349	3.409	2.66 ***	2.183	3.227	2.56 ***	2.096	3.032	0.08	0.96 (0.95, 0.96)
Total Cholesterol (mmol/L)	4.984	4.405	5.621	4.83 ***	4.287	5.478	4.72 ***	4.190	5.355	0.17	0.95 (0.95, 0.96)
Non-HDL Cholesterol (mmol/L)	3.449	2.878	4.095	3.32 **	2.759	3.955	3.22 ***	2.706	3.822	0.25	0.96 (0.96, 0.97)
Remnant Cholesterol (mmol/L)	1.495	1.245	1.779	1.50	1.245	1.789	1.48	1.239	1.770	0.97	0.96 (0.96, 0.96)
VLDL Cholesterol (mmol/L)	0.607	0.463	0.788	0.66 ***	0.491	0.842	0.64 ***	0.489	0.853	0.00 **	0.95 (0.94, 0.95)
LDL Cholesterol (mmol/L)	1.945	1.622	2.317	1.83 ***	1.512	2.174	1.75 ***	1.458	2.060	0.03 *	0.96 (0.95, 0.96)
HDL Cholesterol (mmol/L)	1.506	1.284	1.743	1.46*	1.247	1.696	1.46 **	1.248	1.673	0.32	0.96 (0.96, 0.97)
Triglycerides											
Total Triglycerides (mmol/L)	0.971	0.737	1.318	1.18 ***	0.851	1.690	1.25 ***	0.883	1.797	0.00 ***	0.90 (0.89, 0.91)
Triglycerides in VLDL (mmol/L)	0.647	0.442	0.948	0.84 ***	0.545	1.299	0.89 ***	0.564	1.394	0.00 ***	0.89 (0.88, 0.90)
Triglycerides in LDL (mmol/L)	0.134	0.115	0.156	0.14 *	0.120	0.161	0.14 ***	0.122	0.165	0.19	0.93 (0.92, 0.94)
Triglycerides in HDL (mmol/L)	0.098	0.077	0.124	0.12 ***	0.091	0.144	0.13 ***	0.101	0.157	0.00 **	0.92 (0.92, 0.93)
Phospholipids											
Total Phospholipids in Lipoprotein Particles (mmol/L)	2.965	2.682	3.267	2.97	2.707	3.274	2.98	2.721	3.286	0.71	0.94 (0.94, 0.95)
Phospholipids in VLDL (mmol/L)	0.373	0.276	0.496	0.43 ***	0.307	0.580	0.44 ***	0.306	0.601	0.00 ***	0.93 (0.92, 0.93)
Phospholipids in LDL (mmol/L)	0.668	0.568	0.778	0.63 ***	0.541	0.737	0.61 ***	0.519	0.707	0.09	0.96 (0.95, 0.96)
Phospholipids in HDL (mmol/L)	1.579	1.372	1.794	1.58	1.385	1.794	1.60	1.417	1.816	0.19	0.96 (0.96, 0.96)
Total Lipids											
Total Lipids in Lipoprotein Particles (mmol/L)	9.060	8.039	10.142	9.08	8.086	10.283	9.11	8.012	10.281	0.07	0.94 (0.94, 0.95)
Total Lipids in VLDL (mmol/L)	1.623	1.208	2.205	1.94 ***	1.368	2.681	1.99 ***	1.368	2.804	0.00 ***	0.91 (0.90, 0.92)
Total Lipids in LDL (mmol/L)	2.748	2.310	3.241	2.61 ***	2.188	3.063	2.50 ***	2.109	2.915	0.06	0.96 (0.95, 0.96)
Total Lipids in HDL (mmol/L)	3.188	2.759	3.635	3.15	2.749	3.598	3.19	2.790	3.626	0.23	0.96 (0.96, 0.97)
Lipoprotein Particle Concentrations											
Total Concentration of Lipoprotein Particles (mmol/L)	0.018	0.017	0.020	0.02 ***	0.016	0.019	0.02 ***	0.016	0.019	0.00 **	0.92 (0.91, 0.92)
Concentration of VLDL Particles (mmol/L)	0.000	0.000	0.000	0.00 **	0.000	0.000	0.00 **	0.000	0.000	0.43	0.96 (0.95, 0.96)
Concentration of LDL Particles (mmol/L)	0.001	0.001	0.001	0.00 ***	0.001	0.001	0.00 ***	0.001	0.001	0.24	0.97 (0.96, 0.97)
Concentration of HDL Particles (mmol/L)	0.016	0.015	0.018	0.02 ***	0.015	0.017	0.02 ***	0.014	0.017	0.00 **	0.92 (0.91, 0.93)
Lipoprotein Particle Sizes											
Average Diameter for VLDL Particles (nm)	38.22	37.47	39.13	39.08 ***	37.96	40.32	39.23 ***	37.894	40.732	0.00 ***	0.83 (0.82, 0.85)
Average Diameter for LDL Particles (nm)	23.92	23.85	23.97	23.85 ***	23.77	23.92	23.84 ***	23.738	23.922	0.00 ***	0.53 (0.49, 0.56)
Average Diameter for HDL Particles (nm)	9.694	9.515	9.838	9.72 **	9.543	9.875	9.77 ***	9.582	9.918	0.11	0.98 (0.98, 0.98)
Other Lipids											
Phosphoglycerides (mmol/L)	2.528	2.299	2.771	2.54	2.311	2.787	2.57 **	2.351	2.824	0.57	0.93 (0.93, 0.94)
Ratio of Triglycerides to Phosphoglycerides	0.382	0.300	0.510	0.47 ***	0.354	0.641	0.49 ***	0.359	0.689	0.00 ***	0.90 (0.89, 0.91)
Total Choline’s (mmol/L)	2.872	2.629	3.130	2.86	2.636	3.127	2.87	2.654	3.137	0.39	0.93 (0.92, 0.94)
Phosphatidylcholines (mmol/L)	2.374	2.146	2.613	2.42 **	2.189	2.661	2.47 ***	2.242	2.709	0.72	0.94 (0.94, 0.95)
Sphingomyelins (mmol/L)	0.493	0.447	0.540	0.47 ***	0.431	0.523	0.46 ***	0.423	0.509	0.22	0.92 (0.92, 0.93)
Apolipoproteins											
Apolipoprotein B (g/L)	0.870	0.727	1.023	0.84 *	0.712	0.994	0.83 ***	0.697	0.977	0.43	0.97 (0.96, 0.97)
Apolipoprotein A1 (g/L)	1.534	1.374	1.685	1.50 *	1.362	1.669	1.50 *	1.366	1.652	0.09	0.95 (0.94, 0.95)
Ratio of Apolipoprotein B to Apolipoprotein A1	0.565	0.455	0.691	0.55	0.453	0.687	0.54*	0.446	0.675	0.43	0.96 (0.96, 0.97)
Fatty Acids											
Total Fatty Acids (mmol/L)	12.63	11.28	14.21	13.50 ***	11.79	15.44	13.80 ***	11.903	15.852	0.00 ***	0.84 (0.82, 0.85)
Omega-3 Fatty Acids (mmol/L)	0.536	0.425	0.661	0.57 **	0.447	0.685	0.57 **	0.450	0.685	0.64	0.96 (0.95, 0.96)
Omega-6 Fatty Acids (mmol/L)	5.094	4.660	5.555	5.29 ***	4.805	5.837	5.35 ***	4.835	5.956	0.00 ***	0.81 (0.79, 0.83)
MUFA (mmol/L)	3.035	2.610	3.569	3.69 ***	2.985	4.517	3.95 ***	3.132	4.948	0.00 ***	0.74 (0.72, 0.76)
SFA (mmol/L)	3.915	3.494	4.480	3.90	3.447	4.492	3.87	3.383	4.453	0.09	0.93 (0.93, 0.94)
Amino Acids											
Alanine (mmol/L)	0.324	0.289	0.363	0.35 ***	0.317	0.389	0.39 ***	0.340	0.440	0.00 ***	0.64 (0.61, 0.66)
Glutamine (mmol/L)	0.726	0.675	0.772	0.70 ***	0.651	0.747	0.70 ***	0.653	0.754	0.38	0.78 (0.76, 0.80)
Glycine (mmol/L)	0.251	0.219	0.300	0.23 ***	0.199	0.272	0.23 ***	0.191	0.272	0.99	0.92 (0.91, 0.93)
Histidine (mmol/L)	0.077	0.071	0.082	0.08 ***	0.069	0.080	0.07 ***	0.068	0.079	0.73	0.61 (0.58, 0.64)
Branched-Chain Amino Acids											
Total BCAA (mmol/L)	0.375	0.335	0.424	0.37 *	0.336	0.410	0.38	0.337	0.427	0.52	0.72 (0.70, 0.75)
Isoleucine (mmol/L)	0.048	0.041	0.055	0.05 ***	0.046	0.059	0.06 ***	0.050	0.067	0.00 **	0.51 (0.47, 0.54)
Leucine (mmol/L)	0.110	0.097	0.125	0.10 ***	0.093	0.116	0.10 ***	0.089	0.120	0.55	0.66 (0.63, 0.69)
Valine (mmol/L)	0.218	0.196	0.244	0.21 **	0.196	0.235	0.22	0.198	0.242	0.00 **	0.81 (0.79, 0.83)
Aromatic Amino Acids											
Phenylalanine (mmol/L)	0.062	0.056	0.068	0.06 *	0.056	0.067	0.07 ***	0.060	0.072	0.95	0.60 (0.57, 0.63)
Tyrosine (mmol/L)	0.055	0.049	0.063	0.05 **	0.048	0.061	0.05*	0.048	0.061	0.52	0.68 (0.65, 0.71)
Glycolysis-Related Metabolites											
Glucose (mmol/L)	4.981	4.721	5.269	4.59 ***	4.312	4.892	5.87 ***	5.127	6.665	0.00 ***	0.08 (0.04, 0.12)
Lactate (mmol/L)	1.830	1.619	2.096	1.66 ***	1.491	1.843	1.95 ***	1.687	2.286	0.00 ***	0.31 (0.27, 0.35)
Pyruvate (mmol/L)	0.061	0.053	0.074	0.06 ***	0.048	0.066	0.09 ***	0.069	0.107	0.00 ***	0.20 (0.17, 0.24)
Citrate (mmol/L)	0.064	0.057	0.072	0.06 ***	0.051	0.064	0.06	0.058	0.071	0.00 **	0.55 (0.52, 0.59)
Glycerol (mmol/L)	0.105	0.086	0.129	0.10 ***	0.076	0.124	0.10 ***	0.082	0.123	0.07	0.56 (0.53, 0.59)
Ketone Bodies											
Β-Hydroxybutyrate (mmol/L)	0.113	0.054	0.221	0.07 ***	0.032	0.121	0.01 ***	0.004	0.028	0.00 ***	0.22 (0.18, 0.26)
Acetate (mmol/L)	0.028	0.021	0.038	0.02 ***	0.015	0.028	0.02 ***	0.012	0.024	0.00 ***	0.34 (0.30, 0.38)
Acetoacetate (mmol/L)	0.054	0.031	0.094	0.05 **	0.031	0.078	0.03 ***	0.019	0.036	0.00 ***	0.26 (0.22, 0.31)
Acetone (mmol/L)	0.023	0.017	0.035	0.02 ***	0.015	0.025	0.01 ***	0.012	0.018	0.00 ***	0.53 (0.50, 0.57)
Fluid Balance											
Creatinine (mmol/L)	71.800	64.967	81.013	65.51 ***	59.092	73.864	65.57 ***	59.065	74.096	0.18	0.79 (0.76, 0.80)
Albumin (g/L)	41.887	39.853	44.043	40.86 ***	39.140	42.835	39.87 ***	38.045	41.614	0.00 **	0.76 (0.74, 0.79)
Inflammation											
Glycoprotein Acetyls (mmol/L)	0.845	0.779	0.917	0.83 ***	0.754	0.899	0.81 ***	0.742	0.883	0.79	0.93 (0.92, 0.93)
Lipoprotein Subclass Concentration											
Extremely Large VLDL Particles (mmol/L)	2.42 × 10^−7^	2.11 × 10^−8^	8.19 × 10^−7^	1.43 × 10^−6^ ***	5.32 × 10^−7^	2.97 × 10^−6^	1.67 × 10^−6^ ***	5.84 × 10^−7^	3.68 × 10^−6^	0.00 ***	0.67 (0.64, 0.70)
Very Large VLDL Particles (mmol/L)	2.12 × 10^−6^	1.12 × 10^−6^	3.58 × 10^−6^	2.93 × 10^−6^ ***	1.54 × 10^−6^	5.12 × 10^−6^	3.24 × 10^−6^ ***	1.55 × 10^−6^	5.66 × 10^−6^	0.00 ***	0.89 (0.88, 0.90)
Large VLDL Particles (mmol/L)	7.53 × 10^−6^	4.74 × 10^−6^	1.17 × 10^−5^	9.03 × 10^−6^ ***	5.35 × 10^−6^	1.47 × 10^−5^	9.68 × 10^−6^ ***	5.61 × 10^−6^	1.54 × 10^−5^	0.00 ***	0.92 (0.92, 0.93)
Medium VLDL Particles (mmol/L)	3.26 × 10^−5^	2.45 × 10^−5^	4.25 × 10^−5^	3.49 × 10^−5^ **	2.57 × 10^−5^	4.58 × 10^−5^	3.45 × 10^−5^ **	2.53 × 10^−5^	4.56 × 10^−5^	0.02 *	0.95 (0.94, 0.95)
Small VLDL Particles (mmol/L)	3.43 × 10^−5^	2.65 × 10^−5^	4.42 × 10^−5^	3.48 × 10^−5^	2.71 × 10^−5^	4.45 × 10^−5^	3.41 × 10^−5^	2.68 × 10^−5^	4.31 × 10^−5^	0.00 **	0.93 (0.92, 0.94)
Very Small VLDL Particles (mmol/L)	4.72 × 10^−5^	4.00 × 10^−5^	5.55 × 10^−5^	4.68 × 10^−5^	4.00 × 10^−5^	5.43 × 10^−5^	4.66 × 10^−5^	4.03 × 10^−5^	5.41 × 10^−5^	0.11	0.94 (0.93, 0.95)
IDL Particles (mmol/L)	3.07 × 10^−4^	2.67 × 10^−4^	3.50 × 10^−4^	3.09 × 10^−4^	2.65 × 10^−4^	3.51 × 10^−4^	3.08 × 10^−4^	2.67 × 10^−4^	3.53 × 10^−4^	0.81	0.94 (0.94, 0.95)
Large LDL Particles (mmol/L)	7.65 × 10^−4^	6.40 × 10^−4^	9.12 × 10^−4^	7.24 × 10^−4^ ***	6.07 × 10^−4^	8.61 × 10^−4^	6.96 × 10^−4^ ***	5.88 × 10^−4^	8.16 × 10^−4^	0.00 **	0.95 (0.95, 0.96)
Medium LDL Particles (mmol/L)	3.09 × 10^−4^	2.50 × 10^−4^	3.72 × 10^−4^	2.95 × 10^−4^ ***	2.38 × 10^−4^	3.57 × 10^−4^	2.92 × 10^−4^ ***	2.32 × 10^−4^	3.55 × 10^−4^	0.86	0.95 (0.94, 0.95)
Small LDL Particles (mmol/L)	1.80 × 10^−4^	1.54 × 10^−4^	2.08 × 10^−4^	1.85 × 10^−4^ **	1.56 × 10^−4^	2.15 × 10^−4^	1.81 × 10^−4^	1.52 × 10^−4^	2.17 × 10^−4^	0.00 ***	0.89 (0.88, 0.90)
Very Large HDL Particles (mmol/L)	2.51 × 10^−4^	1.86 × 10^−4^	3.29 × 10^−4^	2.68 × 10^−4^ ***	2.01 × 10^−4^	3.46 × 10^−4^	2.83 × 10^−4^ ***	2.15 × 10^−4^	3.69 × 10^−4^	0.32	0.98 (0.98, 0.98)
Large HDL Particles (mmol/L)	1.70 × 10^−3^	1.10 × 10^−3^	2.29 × 10^−3^	1.74 × 10^−3^	1.14 × 10^−3^	2.35 × 10^−3^	1.80 × 10^−3^ **	1.19 × 10^−3^	2.42 × 10^−3^	0.79	0.98 (0.98, 0.98)
Medium HDL Particles (mmol/L)	4.12 × 10^−3^	3.51 × 10^−3^	4.68 × 10^−3^	3.99 × 10^−3^ *	3.47 × 10^−3^	4.61 × 10^−3^	3.99 × 10^−3^ *	3.49 × 10^−3^	4.54 × 10^−3^	0.02 *	0.95 (0.94, 0.95)
Small HDL Particles (mmol/L)	1.03 × 10^−2^	9.46 × 10^−3^	1.13 × 10^−2^	9.92 × 10^−3^ ***	9.03 × 10^−3^	1.08 × 10^−2^	9.56 × 10^−3^ ***	8.65 × 10^−3^	1.03 × 10^−2^	0.24	0.91 (0.90, 0.92)

^1^ Kruskal–wallis *p*-value is annotated, *** *p* < 0.001, ** *p* < 0.01, * *p* < 0.05, ^2^ Fligner–Killeen *p*-value, *** *p* < 0.001, ** *p* < 0.01, * *p* < 0.05. Abbreviations: BCAA: branched-chain amino acids; CI: confidence intervals; g/L: grams per litre; HDL: high-density lipoprotein; ICC: intra-class correlation coefficients; LDL: low-density lipoprotein; mmol/L: millimoles per litre; MUFA: mono-unsaturated fatty acids; SFA: saturated fatty acids; VLDL: very low-density lipoprotein.

## Data Availability

Data described in the article, code book, and analytic code are held with the Department of Twin Research at King’s College London and will be made available using our normal procedures overseen by the Wellcome Trust and its guidelines as part of our core funding. The application is at: https://twinsuk.ac.uk/resources-for-researchers/access-our-data/, accessed on 11 April 2023.

## Supplementary Materials

The following supporting information can be downloaded at: https://www.mdpi.com/article/10.3390/nu15112638/s1. Table S1: Descriptive characteristics of the PREDICT 1 cohort. Table S2: Characterisation of lipoprotein and metabolite biomarkers. Table S3: Characterisation of postprandial metabolite change stratified by sex.

## Abbreviations

BCAA	branched-chain amino acids
C	cholesterol
CE	cholesterol ester
FC	free cholesterol
GlycA	glycoprotein acetyls
HDL	high-density lipoprotein
HOMA-IR	homeostatic model assessment for insulin resistance
IL-6	interleukin-6
LDL	low-density lipoprotein
ML	machine learning
MUFA	monounsaturated fatty acids
NMR	nuclear magnetic resonance spectroscopy
P	particles
PL	phospholipids
PUFA	polyunsaturated fatty acids
SFA	saturated fatty acids
TG	triacylglycerols
VLDL	very low-density lipoprotein
XXL	XL, L, M, S; extremely large, extra-large, large, medium, small
